# Ionizing radiations induce shared epigenomic signatures unraveling adaptive mechanisms of cancerous cell lines with or without methionine dependency

**DOI:** 10.1186/s13148-021-01199-y

**Published:** 2021-12-01

**Authors:** Youssef Siblini, Céline Chéry, Pierre Rouyer, Jérémie Raso, Amélia Julien, Sébastien Hergalant, Aurélie François, Lina Bezdetnaya, Guillaume Vogin, Jean-Louis Guéant, Abderrahim Oussalah

**Affiliations:** 1 INSERM, UMR_S1256, NGERE (Nutrition, Genetics, and Environmental Risk Exposure), Faculty of Medicine of Nancy, University of Lorraine, 9 Avenue de la Forêt de Haye, 54000 Vandoeuvre-lès-Nancy, Nancy France; 2 Department of Molecular Medicine and Personalized Therapeutics, Department of Biochemistry, Molecular Biology, Nutrition, and Metabolism, University Hospital of Nancy, 54000 Vandoeuvre-lès-Nancy, France; 3 Reference Center for Inborn Errors of Metabolism (ORPHA67872), University Hospital of Nancy, 54000 Vandoeuvre-lès-Nancy, France; 4 Lorraine Institute of Oncology, 54000 Nancy, France; 5 CNRS, UMR_7039, CRAN (Centre de Recherche en Automatique de Nancy), Faculty of Medicine of Nancy, University of Lorraine, 54000 Vandoeuvre-lès-Nancy, France; 6 UMR_7365, IMoPA (Ingénierie Moléculaire Et Ingénierie Articulaire), Faculty of Medicine of Nancy, CNRS-UL, University of Lorraine, 54000 Vandoeuvre-lès-Nancy, France

**Keywords:** Epigenome-wide association study, Epigenome alterations, Aberrant methylation, Radioresistance, Radiation therapy, Ionizing radiation, Metabolic adaptation in cancer, Methionine dependency, Hepatocellular carcinoma, Melanoma, Glioblastoma

## Abstract

**Background:**

Although radiation therapy represents a core cancer treatment modality, its efficacy is hampered by radioresistance. The effect of ionizing radiations (IRs) is well known regarding their ability to induce genetic alterations; however, their impact on the epigenome landscape in cancer, notably at the CpG dinucleotide resolution, remains to be further deciphered. In addition, no evidence is available regarding the effect of IRs on the DNA methylome profile according to the methionine dependency phenotype, which represents a hallmark of metabolic adaptation in cancer.

**Methods:**

We used a case–control study design with a fractionated irradiation regimen on four cancerous cell lines representative of HCC (HepG2), melanoma (MeWo and MeWo-LC1, which exhibit opposed methionine dependency phenotypes), and glioblastoma (U251). We performed high-resolution genome-wide DNA methylome profiling using the MethylationEPIC BeadChip on baseline conditions, irradiated cell lines (cumulative dose of 10 Gy), and non-irradiated counterparts. We performed epigenome-wide association studies to assess the effect of IRs and methionine-dependency-oriented analysis by carrying out epigenome-wide conditional logistic regression. We looked for epigenome signatures at the locus and single-probe (CpG dinucleotide) levels and through enrichment analyses of gene ontologies (GO). The EpiMet project was registered under the ID#AAP-BMS_003_211.

**Results:**

EWASs revealed shared GO annotation pathways associated with increased methylation signatures for several biological processes in response to IRs, including blood circulation, plasma membrane-bounded cell projection organization, cell projection organization, multicellular organismal process, developmental process, and animal organ morphogenesis. Epigenome-wide conditional logistic regression analysis on the methionine dependency phenotype highlighted several epigenome signatures related to cell cycle and division and responses to IR and ultraviolet light.

**Conclusions:**

IRs generated a variation in the methylation level of a high number of CpG probes with shared biological pathways, including those associated with cell cycle and division, responses to IRs, sustained angiogenesis, tissue invasion, and metastasis. These results provide insight on shared adaptive mechanisms of the epigenome in cancerous cell lines in response to IR. Future experiments should focus on the tryptic association between IRs, the initiation of a radioresistance phenotype, and their interaction with methionine dependency as a hallmark of metabolic adaptation in cancer.

**Graphical abstract:**

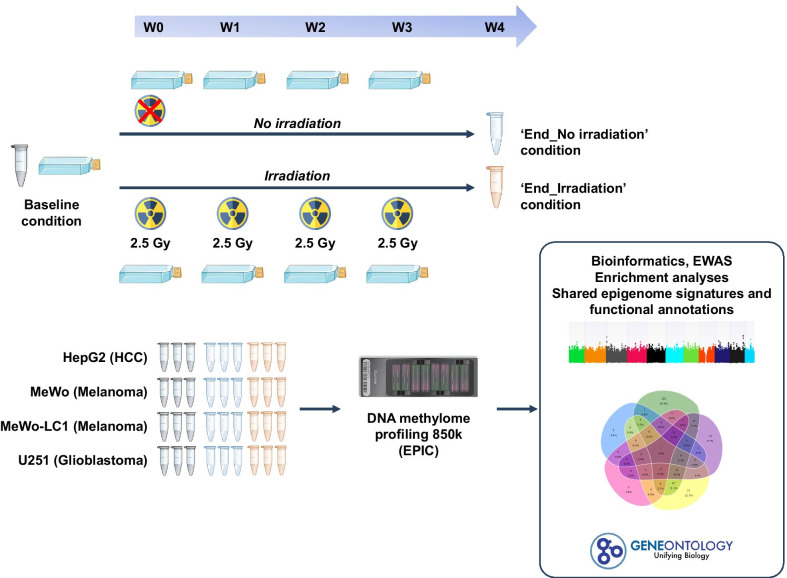

**Supplementary Information:**

The online version contains supplementary material available at 10.1186/s13148-021-01199-y.

## Introduction

Epigenome alterations, including modifications of DNA methylation, represent a hallmark of cancer initiation, progression, metastasis, and recurrence [1–5]. Cancerous cell lines use epigenomic reprogramming to develop new strategies against anticancer treatments, including radiation therapy [1]. DNA methylation requires S-adenosyl methionine (SAM) as a methyl donor for which methionine represents the immediate metabolic precursor. Dependency to methionine, also called methionine dependency, is a metabolic adaptation that occurs in association with cancerous transformation, and its interaction with epigenetic modifications is increasingly recognized as a driver of tumorigenesis [2, 4, 6–8]. In this context, epigenetic modifications have been hypothesized as key drivers for tumor aggressiveness, with resistant phenotype to conventional chemo- and radiotherapy and an ability to develop metastases [5].

Radiation therapy represents a core modality of cancer treatment [9]. For instance, it is indicated in newly diagnosed or recurrent glioblastoma [10–12] and has gained interest in recent years in the therapeutic algorithm of hepatocellular carcinoma (HCC), notably through stereotactic body radiation therapy [13–16]. In the case of cutaneous malignant melanoma, radiation therapy is not recommended in current guidelines; however, numerous studies reported its efficacy, notably in the setting of elective adjuvant irradiation for eradicating subclinical nodal metastases [17]. Radioresistance is a leading cause of cancer progression [18]. Ionizing radiations (IRs) are well known for their ability to induce cellular stress with subsequent alterations in several biological functions, including cellular stress response, cell signaling, DNA synthesis and repair, cell cycle and differentiation, and cell adhesion [19–24].

The effect of IRs is well known regarding their ability to induce genetic alterations [25]. Besides, epigenome alterations have been hypothesized as a potential contributing factor to radioresistance, notably through DNA methylation, histone modification, and chromatin remodeling, which may induce transcriptional reprogramming that enables cells to avoid IR effects [18, 26, 27]. Rodent animal models have shown that IRs affect DNA and histones’ methylation in several organs such as the liver, bone marrow, thymus, and spleen [28]. However, the effect of IRs on epigenetic alterations in cancer, notably at a nucleotide resolution level (CpG dinucleotide), remains to be further deciphered [25].

To date, data are sparse regarding the effect of IRs on the epigenomic landscape of established cancerous cell lines, notably following a fractionated irradiation regimen. Moreover, no work has systematically evaluated the impact of IRs at a nucleotide resolution level using a standardized protocol of fractionated irradiation using multiple cancerous cell lines. In addition, no data have been reported regarding specific epigenome signatures, considering the methionine dependency phenotype, which represents a hallmark of metabolic adaptation in cancer [6–8].

To address this knowledge gap, we used four cancerous cell lines to infer potential epigenomic alterations that IRs could induce in HCC, glioblastoma, and melanoma. HCC and glioblastoma were used as an example of cancer treatable by IRs and melanoma as an example of cancer with no indication of IR according to current guidelines. We were able to identify shared annotation pathways for epigenomic signatures associated with and exposure to IR. Furthermore, we performed epigenome-wide conditional analyses according to methionine dependency phenotype and highlighted several epigenome signatures related to cell division and response to IR.

## Methods

### Design of the EpiMet project, oversight, and study aims

The EpiMet project was designed to assess the effect of IR, as a cellular stress model, on the epigenome landscape of cancerous cell lines using an EWAS approach. We also looked for potentially contrasted epigenome signatures considering the methionine dependency phenotype using well-phenotyped cell lines. For this purpose, we used four cell lines and three cancer models: HCC, melanoma, and glioblastoma. The EpiMet project was declared at the University of Lorraine (Research Pole of Biology, Medicine, and Health Sciences) and registered under the ID #AAP-BMS_003_211.

### Cell lines and cell culture procedures

We cultivated the human HCC HepG2 (ATCC®) cell lines in Dulbecco’s modified Eagle’s medium (DMEM) (Sigma–Aldrich, Saint-Quentin-Fallavier, France) supplemented with 10% volume per volume of heat-inactivated fetal bovine serum (FBS) (Sigma–Aldrich), 1% glutamine (Sigma–Aldrich), and 1% penicillin–streptomycin (P/S) (Sigma–Aldrich). We cultivated the human melanoma cell lines (MeWo [ATCC®], MeWo-LC1 [Dr. Robert Liteplo, University of Ottawa, Ottawa, Ontario]) in DMEM supplemented with 10% volume per volume of FBS, 2% glutamine, and 1% P/S. We cultivated the human glioblastoma cell line U251 (European Collection of Authenticated Cell Cultures [ECACC]) in DMEM supplemented with 10% volume per volume of heat-inactivated FBS, 1% non-essential amino acid (NEAA) (Sigma-Aldrich), 1% glutamine, 1% pyruvate (Sigma–Aldrich), and 1% P/S. All the cells were incubated in a humidified atmosphere with 5% CO_2_ at 37 °C.

### Experimental design, cell line irradiation, and cell material

We used a case–control study design with a fractionated irradiation regimen on four cancerous cell lines. In the irradiation arm, the cells were exposed to four IR doses at 2.5 Gy per irradiation at a one-week interval, totaling a cumulative dose of 10 Gy. In the control arm, cell lines were not irradiated and were maintained in similar conditions for the same study period. All cells were maintained in standard culture conditions with a renewal of culture media two times a week. To perform DNA methylome profiling, we prepared flash-frozen cell pellets from irradiated cell lines (‘End_Irradiation’) one week after the fourth irradiation and their non-irradiated counterparts (‘End_No irradiation’) on the same day. We also prepared flash-frozen cell pellets from baseline conditions for each cell line (‘Baseline’) before the initiation of the study. All experiments were performed in biological triplicate, totaling 36 cell flasks (nine flasks per cell line: three Baseline, three End_Irradiation, three End_No irradiation). We performed cell line irradiation at the Department of Radiation Oncology of the Lorraine Institute of Oncology (Vandoeuvre-lès-Nancy, France). The cells were irradiated in the plateau growth phase to avoid any artifacts resulting from the cell cycle. Irradiation was performed on a 6-MeV X-ray linear accelerator (Clinac 2100, Varian, Palo Alto, USA) (see Graphical Abstract).

### DNA methylome analyses

We carried out bisulfite conversion of 600 ng of DNA extracted from cell lines using the EZ DNA Methylation kit (Zymo Research, Proteigene, Saint-Marcel, France). The genome-wide profiling of DNA methylome was determined using the Infinium MethylationEPIC BeadChip array (Illumina, Paris, France) at the Functional Genomics Facility of the INSERM unit UMR_S 1256 (NGERE, Vandoeuvre-lès-Nancy, France). The Infinium MethylationEPIC BeadChip provides coverage of > 850,000 CpG probes in enhancer regions, gene bodies, promoters, and CpG islands. The arrays were scanned on an Illumina iScan® system, and raw methylation data were extracted using Illumina’s Genome Studio methylation module. For each CpG probe, the methylation level was described as a *β* value, ranging between 0 (fully unmethylated CpG probe) and 1 (fully methylated CpG probe). Background correction and normalization were implemented using the SWAN method (R Package Minfi) [29]. Probe annotation information, including sequence and chromosome location for the Infinium MethylationEPIC BeadChip array, was retrieved from the Infinium MethylationEPIC v1.0 B5 Manifest File.

### Quality controls of methylome array data and bioinformatics analyses

We visually inspected the whole-genome distribution of the CpG probes according to their *β* value. We performed primary component analysis (PCA) to assess the clustering of methylation profiles according to the whole methylation landscape of the studied cell lines. The top ten principal components (eigenvectors, EV) were calculated with their respective eigenvalue. PCA plots were used to report on the three top eigenvalues. We performed our analytical approach using a sequential approach. In step #1, we compared the epigenome landscape between ‘Baseline’ and ‘End_No irradiation’ conditions to assess whether the maintenance of cell lines affected DNA methylation. In step #2, we performed an EWAS that pooled ‘Baseline’ and ‘End_No irradiation’ conditions to compare them with the ‘End_Irradiation’ condition. The aim of step #2 was to assess whether the exposure to a cumulative dose of 10 Gy affected the DNA methylation landscape of the studied cell lines. In this step, we performed a sensitivity analysis EWAS by removing the ‘Baseline’ condition from the control group to compare the ‘End_Irradiation’ versus ‘End_No irradiation’ condition. In step #3, we performed the same analysis described in step #2 on each of the four cell lines. Finally, in step #4, we assessed using enrichment analyses the gene ontology (GO) pathways significantly associated with exposure to IRs and looked for shared GO annotations between the studied cell lines. In each EWAS (steps #1–3), we compared the mean *β* values of each CpG probe between the two subgroups using the *t*-test with Bonferroni correction. Output data included the mean *β* values in each subgroup, the difference of *β* values, the nominal *P*-value, and the Bonferroni corrected *P*-value. To assess the effect of IRs on the epigenome landscape with respect to the methionine dependency phenotype, we performed conditional logistic regression EWAS using the methylome profiles from methionine-dependent cell lines (MeWo-LC1) and their methionine-independent counterpart (MeWo) [30]. We used the ‘End_Irradiation’ as the dependent variable and the methionine-dependent phenotype as the conditional covariate. EWAS results were reported using an epi-Manhattan plot. We performed enrichment analyses on CpG probes exhibiting increased or decreased methylation signatures (difference of *β* values > 0.1 or <  − 0.1, respectively) using the GO Enrichment Analysis tool [31]. For the conditional logistic regression EWAS, we performed enrichment analyses on the CpG probes with beta regression coefficient values > 200 or <  − 200. We used PANTHER ‘GO-Slim Biological Process’ and ‘GO-biological process complete’ annotation datasets and the REViGO tool to summarize and visualize statistically significant GO terms based on their calculated metrics (uniqueness and dispensability) [32]. We identified top enriched GO annotations using quantile–quantile plot representation. All statistical analyses were performed using the SNP and Variation Suite (v8.8.1; Golden Helix, Inc., Bozeman, MT, USA) and MedCalc, version 19.5.3 (MedCalc Software, Ostend, Belgium).

## Results

### Principal component analyses for DNA methylome profile assessment

**Fig. 1.**
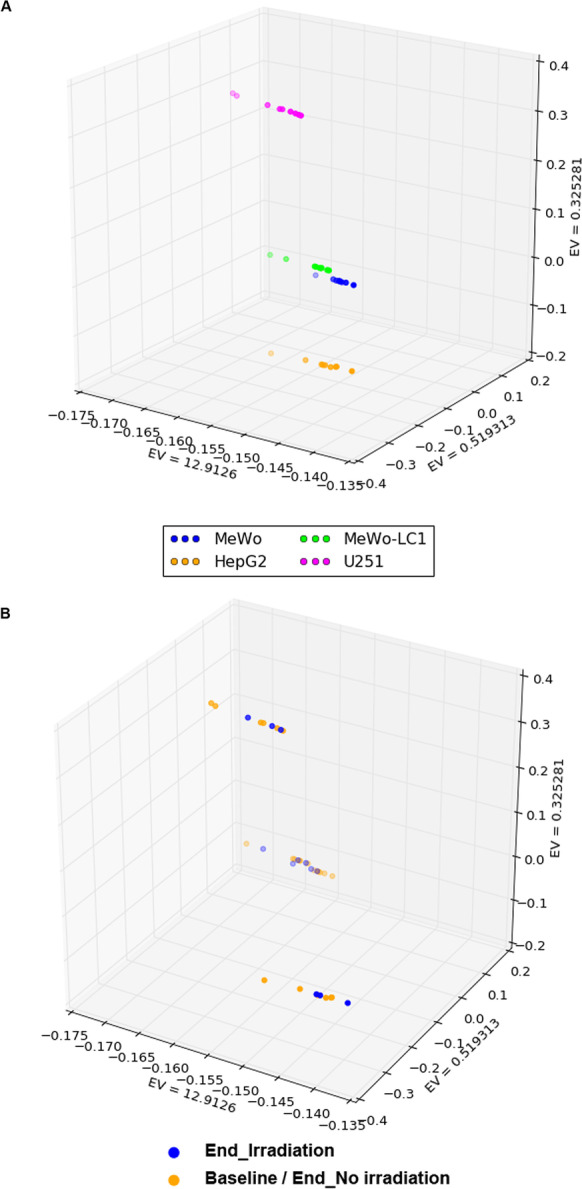
3-D plot using the three top eigenvectors (EV1, EV2, EV3) derived from the primary component analysis on the genome-wide methylome landscape of the studied cell lines. Panel (**A**) reports the clustering per cell line; panel (**B**) reports the clustering according to the two study conditions: ‘Baseline/End_No irradiation and’ ‘End_Irradiation’

We performed DNA methylome profiling of the 36 samples corresponding to nine flasks (three samples of the conditions ‘Baseline,’ ‘End_Irradiation,’ and ‘End_No irradiation’) for each of the four studied cell lines. All methylome profiles passed quality checks and exhibited a valid *β* value density distribution (Additional file 1: Figure S1). In PCA on genome-wide DNA methylome profiles, we found a clustered distribution according to the cell line type (Fig. 1A). Cell line clustering was associated with the third PCA vector (EV = 0.32). In both 3-D and 2-D visual inspection of the PCA plots, we found no systematic clustering of DNA methylome profiles between ‘Baseline’ and ‘End_No irradiation’ conditions (Additional file 2: Figure S2, Additional file 3: Figure S3). Furthermore, we found no significant clustering of DNA methylome profiles according to the irradiation status (Fig. 1B, Additional file 3: Figure S3).

### Epigenome-wide association studies

**Fig. 2:**
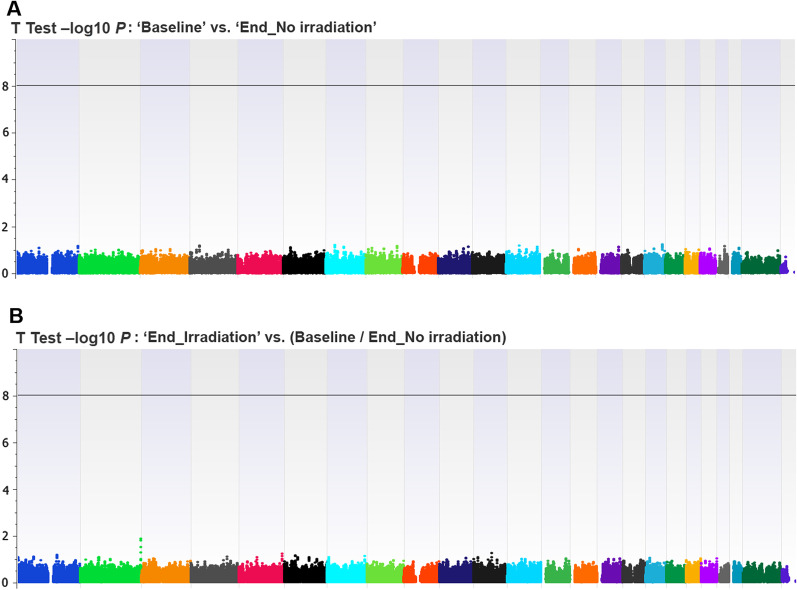
Epi-Manhattan plot reporting the results of the epigenome-wide association study that compared ‘Baseline’ versus ‘End_No irradiation’ (panel **A**) and ‘End_Irradiation’ versus (Baseline/End_No irradiation) (panel **B**)

**Fig. 3:**
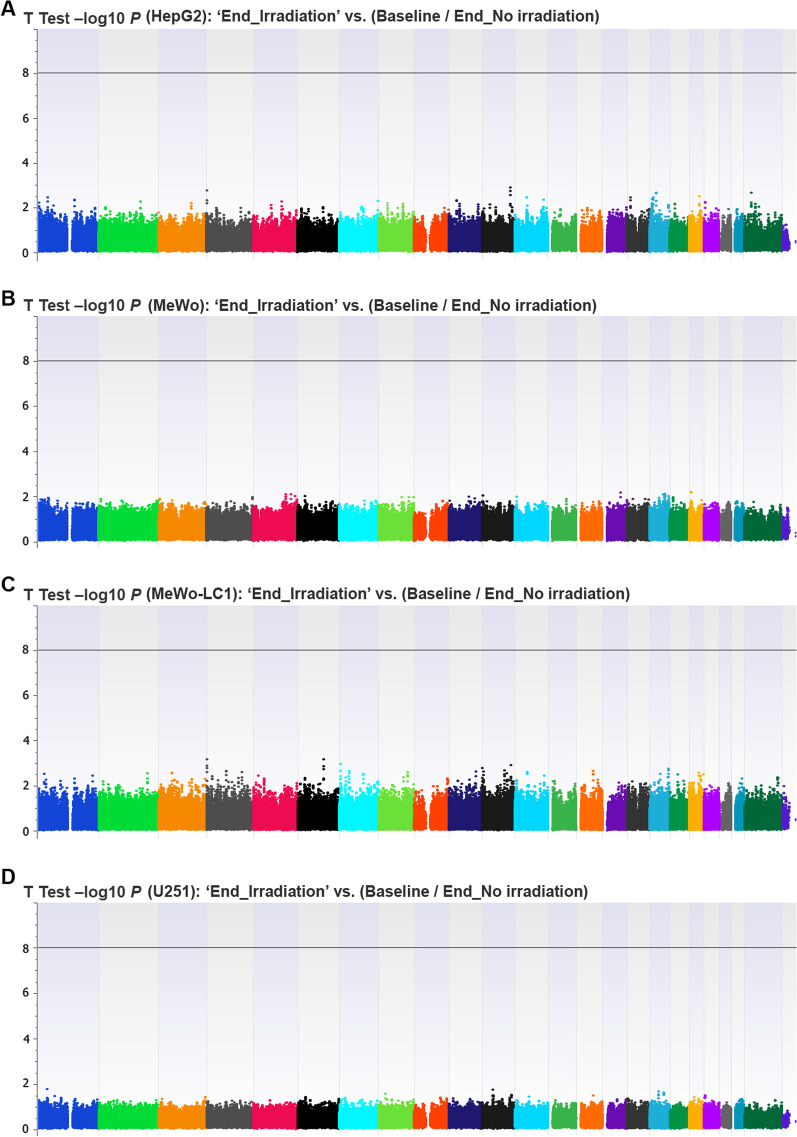
Epi-Manhattan plot reporting the results of the epigenome-wide association study that compared ‘End_Irradiation’ versus (Baseline/End_No irradiation) in HepG2 (panel **A**), MeWo (panel **B**), MeWo-LC1 (panel **C**), and U251 (panel **D**) cell lines

**Fig. 4:**
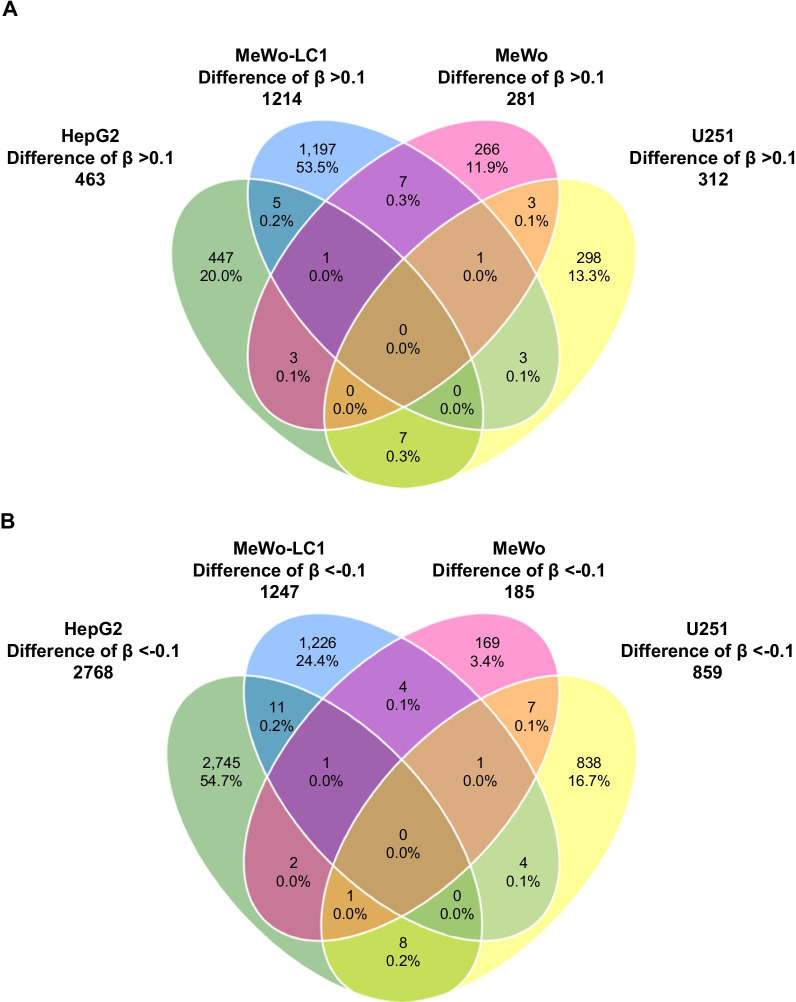
Venn diagram illustrating the shared CpG probes with an increased (panel **A**) or decreased (panel **B**) DNA methylome signature between HepG2, MeWo, MeWo-LC1, and U251 cell lines

We found no statistically significant locus or CpG probe in the EWAS that compared ‘Baseline’ and ‘End_No irradiation’ conditions (Fig. 2A). This allowed us to pool both conditions and compare them with the ‘End_Irradiation’ condition in the whole and subgroup analyses according to cell line type. In the EWAS that assessed the effect of IRs on the DNA methylation profile (End_Irradiation vs. [End_No irradiation/Baseline]), we found no statistically significant locus or CpG probe (Fig. 2B, Additional file 4: Table S1a). In sensitivity analysis EWAS, we found consistent results by comparing the ‘End_Irradiation’ versus ‘End_No irradiation’ condition (Additional file 5: Table S1b). In EWAS subanalyses that assessed the effect of IRs on individual cell types, we found no statistically significant CpG (Fig. 3, Additional file 6: Table S2, Additional file 7: Table S3, 8: Table S4, Additional file 9: Table S5). When we compared the EWAS subanalyses, we found no shared CpG probes exhibiting a difference of *β* values > 0.1 (Fig. 4A) or <  − 0.1 (Fig. 4B) between the four cell lines. In the conditional logistic regression EWAS that looked for specific epigenome signatures considering the methionine dependency phenotype, we found no statistically significant locus or CpG probe at the genome-wide level (Additional file 10: Figure S4).

**Table 1:** Shared gene ontology annotations for enrichment analyses on CpG probes that exhibited a *β* values difference > 0.1 before and after ionizing radiation of HepG2 and MeWo-LC1 cell lines

GO biological process complete	HepG2*			MeWo-LC1*		
	FE	*P*-value	FDR, *P*-value	FE	*P*-value	FDR, *P*-value
Blood circulation (GO:0008015)	3.22	2.22 × 10^–5^	2.34 × 10^–2^	2.33	7.49 × 10^–5^	1.62 × 10^–2^
Animal organ morphogenesis (GO:0009887)	2.34	1.19 × 10^–5^	1.71 × 10^–2^	1.97	1.58 × 10^–6^	8.04 × 10^–4^
Plasma membrane-bounded cell projection organization (GO:0120036)	2.26	3.86 × 10^–6^	7.63 × 10^–3^	1.73	2.98 × 10^–5^	8.27 × 10^–3^
Cell projection organization (GO:0030030)	2.23	5.33 × 10^–6^	8.42 × 10^–3^	1.72	2.86 × 10^–5^	8.07 × 10^–3^
Cell development (GO:0048468)	1.95	2.02 × 10^–5^	2.45 × 10^–2^	2.06	3.40 × 10^–12^	5.96 × 10^–9^
Anatomical structure morphogenesis (GO:0009653)	1.94	7.92 × 10^–7^	2.50 × 10^–3^	1.82	1.49 × 10^–10^	1.96 × 10^–7^
Nervous system development (GO:0007399)	1.86	4.60 × 10^–6^	8.08 × 10^–3^	1.94	1.67 × 10^–13^	3.31 × 10^–10^
Cell differentiation (GO:003054)	1.58	1.90 × 10^–5^	2.51 × 10^–2^	1.75	9.46 × 10^–16^	2.99 × 10^–12^
Cellular developmental process (GO:0048869)	1.58	2.13 × 10^–5^	2.40 × 10^–2^	1.75	6.11 × 10^–16^	2.41 × 10^–12^
System development (GO:0048731)	1.56	2.62 × 10^–6^	6.89 × 10^–3^	1.64	8.72 × 10^–15^	1.97 × 10^–11^
Multicellular organism development (GO:0007275)	1.55	4.60 × 10^–7^	1.82 × 10^–3^	1.61	5.24 × 10^–16^	2.76 × 10^–12^
Anatomical structure development (GO:0048856)	1.55	1.51 × 10^–7^	1.19 × 10^–3^	1.59	9.01 × 10^–17^	7.12 × 10^–13^
Developmental process (GO:0032502)	1.51	2.22 × 10^–7^	1.17 × 10^–3^	1.59	5.53 × 10^–19^	8.74 × 10^–15^
Multicellular organismal process (GO:0032501)	1.45	1.10 × 10^–7^	1.73 × 10^–3^	1.46	1.64 × 10^–15^	4.33 × 10^–12^

FE: fold enrichment; DFR: false discovery rate

* No significant GO annotation was found for the CpG probes that exhibited a β values difference > 0.1 before and after ionizing radiation for MeWo and U251 cell lines

### Enrichment analyses

**Fig. 5:**
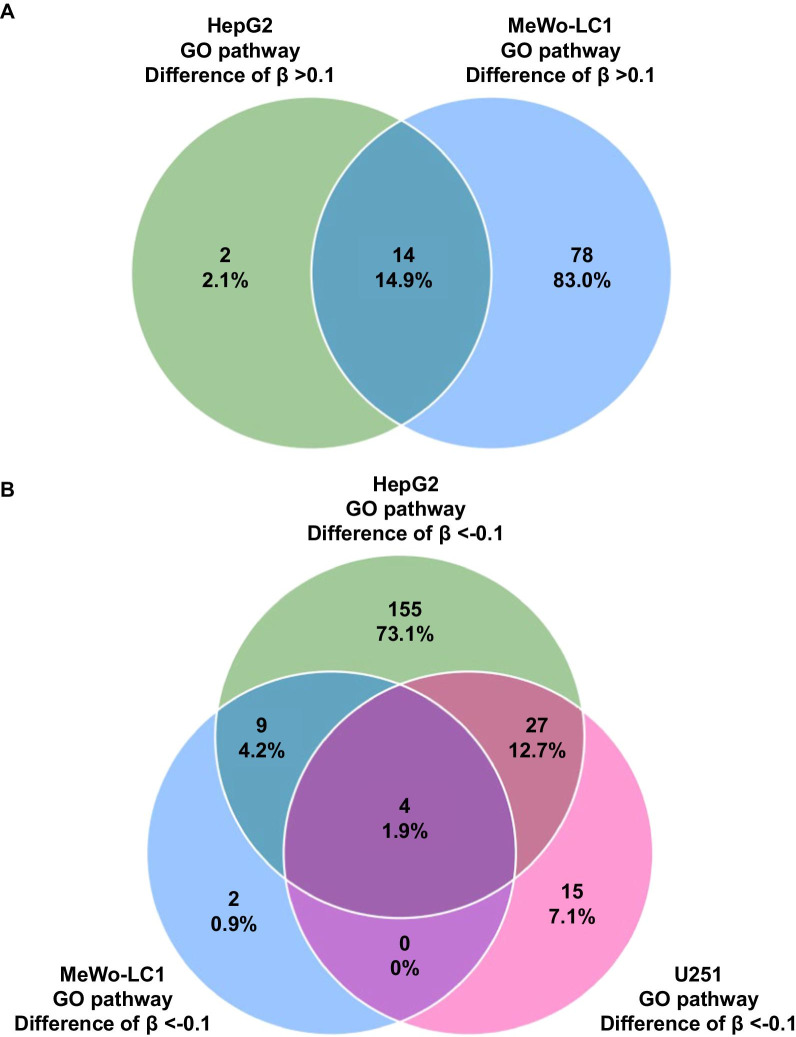
**A** Venn diagram illustrating the shared GO annotation pathways between HepG2 and MeWo-LC1 cell lines in association with CpG probes with an increased methylation level; **B** Venn diagram illustrating the shared GO annotation pathways between HepG2, MeWo-LC1, and U251 cell lines in association with CpG probes with a decreased methylation level

**Fig. 6:**
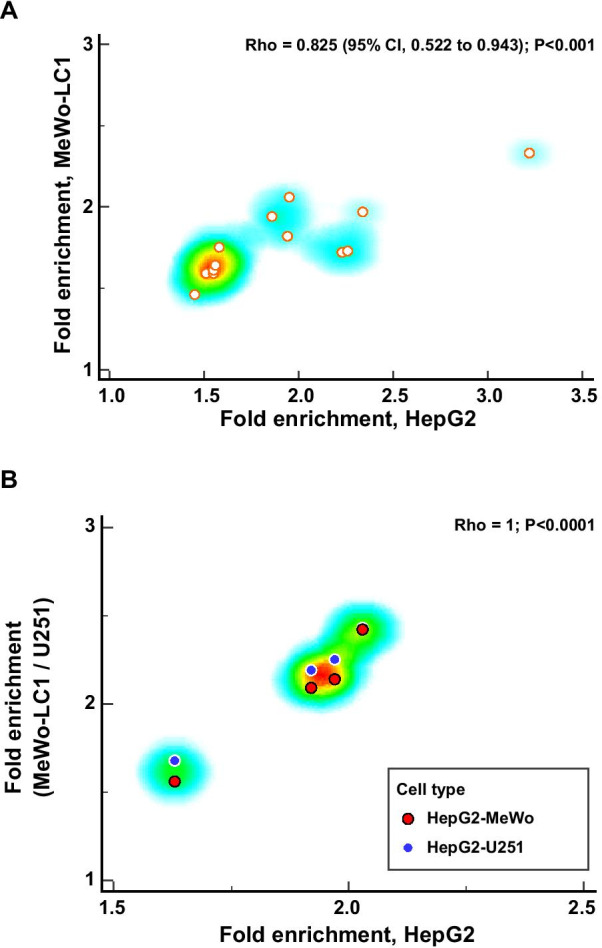
**A** Correlation plot reporting the enrichment folds of the shared gene ontology annotation pathways between HepG2 and MeWo-LC1 cell lines for the CpG probes that exhibited a *β* values difference > 0.1 before and after ionizing radiation (no significant gene ontology annotation was found for the CpG probes that exhibited a *β* values difference > 0.1 before and after ionizing radiation for MeWo and U251 cell lines); **B** correlation plot reporting the enrichment folds of the shared gene ontology annotation pathways between HepG2, MeWo-LC1, and U251 cell lines for the CpG probes that exhibited a *β* values difference <  − 0.1 before and after ionizing radiation (no significant gene ontology annotation was found for the CpG probes that exhibited a *β* values difference <  − 0.1 before and after ionizing radiation for MeWo cell line)

Enrichment analyses that used the CpG probes exhibiting a difference of *β* values > 0.1 or <  − 0.1 found no statistically significant GO annotation when all cell lines were considered together (Additional file 11: Table S6a,b). However, when cell lines were considered separately, we found significantly enriched GO annotations on CpG probes that exhibited a β values difference > 0.1 (increase in methylation level) among HepG2 and MeWo-LC1 cell lines (Fig. 5A and Additional file 11: Table S6c–f) and for CpG probes that exhibited a *β* values difference <  − 0.1 (decrease in methylation level) among HepG2, MeWo-LC1, and U251 cell lines (Fig. 5B and 11: Table S6g–j). Importantly, we found 14 shared GO annotation pathways between HepG2 and MeWo-LC1 cell lines in association with CpG probes with an increased methylation level (Fig. 5A and Additional file 12: Figure S5) with significantly correlated enrichment folds (Spearman’s coefficient of rank correlation [rho] = 0.825 (95% CI, 0.522 to 0.943); *P* < 0.001) for the following main annotations: blood circulation, plasma membrane-bounded cell projection organization, and cell projection organization (Table 1; Fig. 6A). Similarly, we found mirrored results with shared GO annotation pathways between HepG2, MeWo-LC1, and U251 cell lines in association with CpG probes with a decreased methylation level (Fig. 5B and Additional file 12: Figure S5) with significantly correlated enrichment folds (rho = 1; *P* < 0.0001) (Fig. 6B), including regulation of plasma membrane-bounded cell projection organization and regulation of cell projection organization (Table 2).

**Table 2:** Shared gene ontology annotations for enrichment analyses on CpG probes that exhibited a *β* values difference < − 0.1 before and after ionizing radiation of HepG2, MeWo-LC1, and U251 cell lines

GO biological process complete	HepG2*			MeWo-LC1*			U251*		
	FE	*P*-value	FDR, *P*-value	FE	*P*-value	FDR, *P*-value	FE	*P*-value	FDR, *P*-value
Regulation of neuron projection development (GO:0010975)	2.03	4.69 × 10^–6^	1.35 × 10^–3^	2.42	1.05 × 10^–5^	1.84 × 10^–2^	2.43	6.78 × 10^–5^	3.25 × 10^–2^
Regulation of plasma membrane-bounded cell projection organization (GO:0120035)	1.97	2.76 × 10^–7^	1.25 × 10^–4^	2.14	1.58 × 10^–5^	2.28 × 10^–2^	2.25	2.07 × 10^–5^	1.72 × 10^–2^
Regulation of cell projection organization (GO:0031344)	1.92	6.05 × 10^–7^	2.33 × 10^–4^	2.09	3.50 × 10^–5^	4.25 × 10^–2^	2.19	4.32 × 10^–5^	2.53 × 10^–2^
Nervous system development (GO:0007399)	1.63	6.94 × 10^–12^	1.22 × 10^–8^	1.56	1.22 × 10^–5^	1.93 × 10^–2^	1.68	1.76 × 10^–6^	2.53 × 10^–3^

FE: fold enrichment; DFR: false discovery rate

*No significant GO annotation was found for the CpG probes that exhibited a β values difference < −0.1 before and after ionizing radiation for MeWo cell line

**Fig. 7:**
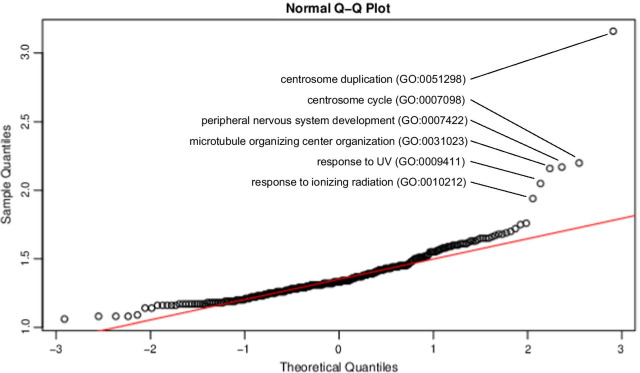
Q–Q plot for the top enriched gene ontology annotations on CpG probes with beta regression coefficient values > 200 in association with ionizing radiations using conditional logistic regression EWAS on the methionine dependency phenotype

**Fig. 8:**
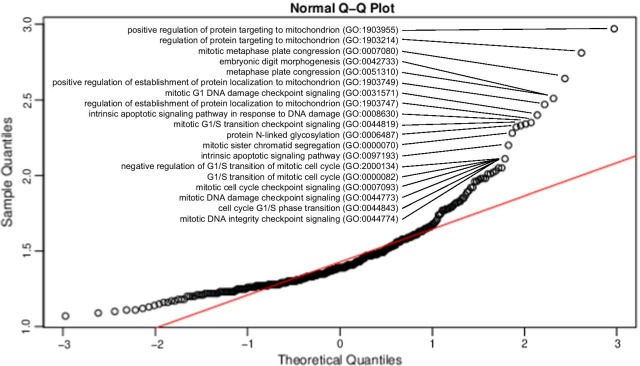
Q–Q plot for the top enriched gene ontology annotations on CpG probes with beta regression coefficient values <  − 200 in association with ionizing radiations using conditional logistic regression EWAS on the methionine dependency phenotype

In conditional logistic regression EWAS that compared methionine-dependent MeWo-LC1 cells to their non-methionine dependent mother cells MeWo, the enrichment analyses performed on the CpG probes with beta regression coefficient values > 200 highlighted several pathways, including cell division and responses to IR and ultraviolet light (Table 3, Fig. 7, and Additional file 13: Table S7 a). Conversely, enrichment analyses performed on the CpG probes with beta regression coefficient values <  − 200 highlighted pathways related to cell cycle, cell division, and DNA damage (Table 4, Fig. 8, and Additional file 13: Table S7 b).

**Table 3:** Top enriched gene ontology annotations on CpG probes with beta regression coefficient values > 200 in association with ionizing radiations using conditional logistic regression EWAS on the methionine dependency phenotype

GO biological process complete	CLR-EWAS for the methionine dependency phenotype (MeWo-LC1 vs. MeWo)		
	FE*	*P*-value	FDR, *P*-value
Centrosome duplication (GO:0051298)	3.16	1.05 × 10^–4^	7.80 × 10^–3^
Centrosome cycle (GO:0007098)	2.20	2.11 × 10^–4^	1.41 × 10^–2^
Peripheral nervous system development (GO:0007422)	2.17	4.85 × 10^–4^	2.83 × 10^–2^
Microtubule organizing center organization (GO:0031023)	2.16	1.22 × 10^–4^	8.91 × 10^–3^
response to UV (GO:0,009,411)	2.05	1.53 × 10^–5^	1.47 × 10^–3^
Response to ionizing radiation (GO:0010212)	1.94	1.00 × 10^–4^	7.49 × 10^–3^

CLR: conditional logistic regression; FE: fold enrichment; DFR: false discovery rate

* Top significantly enriched GO annotations were defined using a Q–Q plot ranking of fold enrichment values. The full list of significantly enriched gene ontology annotations is reported in Additional file 13: Table S7a

**Table 4:** Top enriched gene ontology annotations on CpG probes with beta regression coefficient values < − 200 in association with ionizing radiations using conditional logistic regression EWAS on the methionine dependency phenotype

GO biological process complete	CLR-EWAS for the methionine dependency phenotype (MeWo-LC1 vs. MeWo)		
	FE*	*P*-value	FDR, *P*-value
Positive regulation of protein targeting to mitochondrion (GO:1903955)	3.00	1.03 × 10^–3^	4.57 × 10^–2^
Regulation of protein targeting to mitochondrion (GO:1903214)	2.80	3.50 × 10^–4^	1.87 × 10^–2^
Mitotic metaphase plate congression (GO:0007080)	2.60	5.84 × 10^–4^	2.87 × 10^–2^
Embryonic digit morphogenesis (GO:0042733)	2.51	4.70 × 10^–4^	2.40 × 10^–2^
Metaphase plate congression (GO:0051310)	2.47	5.38 × 10^–4^	2.67 × 10^–2^
Positive regulation of establishment of protein localization to mitochondrion (GO:1903749)	2.40	9.12 × 10^–4^	4.19 × 10^–2^
Mitotic G1 DNA damage checkpoint signaling (GO:0031571)	2.35	4.65 × 10^–4^	2.38 × 10^–2^
Regulation of establishment of protein localization to mitochondrion (GO:1903747)	2.30	3.61 × 10^–4^	1.91 × 10^–2^
Intrinsic apoptotic signaling pathway in response to DNA damage (GO:0008630)	2.30	6.77 × 10^–4^	3.24 × 10^–2^
Mitotic G1/S transition checkpoint signaling (GO:0044819)	2.30	5.24 × 10^–4^	2.62 × 10^–2^
Protein N-linked glycosylation (GO:0006487)	2.30	6.84 × 10^–4^	3.25 × 10^–2^
Mitotic sister chromatid segregation (GO:0000070)	2.20	4.52 × 10^–5^	3.31 × 10^–3^
Intrinsic apoptotic signaling pathway (GO:0097193)	2.11	1.65 × 10^–5^	1.43 × 10^–3^
Negative regulation of G1/S transition of mitotic cell cycle (GO:2000134)	2.05	3.93 × 10^–4^	2.05 × 10^–2^
G1/S transition of mitotic cell cycle (GO:0000082)	2.05	6.21 × 10^–4^	3.00 × 10^–2^
Mitotic cell cycle checkpoint signaling (GO:0007093)	2.03	2.85 × 10^–5^	2.26 × 10^–3^
Mitotic DNA damage checkpoint signaling (GO:0044773)	2.02	3.85 × 10^–4^	2.02 × 10^–2^
Cell cycle G1/S phase transition (GO:0044843)	2.01	9.98 × 10^–4^	4.47 × 10^–2^
Mitotic DNA integrity checkpoint signaling (GO:0044774)	2.01	3.16 × 10^–4^	1.73 × 10^–2^

CLR: conditional logistic regression; FE: fold enrichment; DFR: false discovery rate

*Top significantly enriched GO annotations were defined using a Q–Q plot ranking of fold enrichment values. The full list of significantly enriched gene ontology annotations is reported in Additional file 13: Table S7b

## Discussion

In the EpiMet study, we assessed the effect of a fractionated regimen of IRs on the epigenome landscape of cancerous cell lines representative of leading causes of cancer incidence and mortality worldwide, including HCC, cutaneous melanoma, and glioblastoma, with a cumulative incidence of over one million new cases diagnosed in 2020 [33]. HCC is the most common primary malignant tumor of the liver [34–36], glioblastoma is the most common and most aggressive malignant primary brain tumor [37], and cutaneous malignant melanoma has an increasing incidence worldwide.

Using an original approach through a case–control study design and four cancerous cell lines (HepG2, MeWo, MeWo-LC1, and U251), we provided better insight at a high-resolution scale on the DNA methylome variations in association with IRs. While we did not find monogenic epigenome signatures in association with exposure to IRs, we identified significant enrichments in CpG probes exhibiting increased or decreased methylation levels (difference of *β* values > 0.1 or <  − 0.1, respectively) in association with exposure to IRs that were consistent with epigenomic adaptive mechanisms involving sustained angiogenesis, tissue invasion, and metastasis. The enrichment folds for the shared epigenomic signatures were found to be highly correlated across cell lines. In addition, we highlighted several epigenome signatures related to cell division and response to IRs in association with the methionine dependency phenotype that we addressed using two melanoma cell lines with opposite methionine dependency phenotypes.

Despite unaltered DNA sequences, epigenetic alterations can alter gene expression and cell functions [38, 39]. DNA methylation alterations represent a dynamic process described under physiological and pathological conditions, including aging, cancer, complex phenotype diseases, and inherited disorders [40–42]. IRs represent a well-defined experimental model of cellular stress [19–24]. DNA methylation has been shown to vary in response to cellular stress conditions [43–45]. Mitochondrial dysfunction, induced by cellular stress, triggers a methylation-dependent pro-survival response with enhanced DNA methylation of tumor suppressor genes and pathways involved in cell survival regulation [45].

Few studies have assessed the effect of IRs on cancerous cell lines, and no study assessed the methylome landscape using the high-density EPIC BeadChip for methylome profiling. A study on the MDA-MB-231 cell line corresponding to metastatic mammary adenocarcinoma assessed the effect of IRs at a unique irradiation dose of 2 or 6 Gy [46]. The DNA methylome profiling assessed by the Infinium HumanMethylation450 BeadChip (M450k array) demonstrated enrichment in GO annotations related to cell cycle, DNA repair, and apoptosis pathways [46]. A comparative analysis of the methylome profile of radiosensitive (SCC-61) and radioresistant (rSCC-61) human squamous cell carcinoma cell lines (head and neck squamous cell cancer) reported an enrichment in glucocorticoid receptor signaling, fatty acid α-oxidation, and cell cycle regulation as top canonical pathways associated with radiation resistance [47]. A recent study investigated the DNA methylation alterations following IRs of glioma stem cells using the M450k array after repeated doses of 2 or 4 Gy using two regimens of 3 or 15 fractions [48]. The DNA methylome signatures were assessed 14 days after the last irradiation. No significant DNA methylation alterations were observed in cell lines that received few fractions of radiations [48]. Conversely, higher radiation doses induced a variation in DNA methylation level at numerous CpG sites whose annotations potentially reflect a cellular response to radiation stress [48].

To date, no study has assessed the DNA methylome alterations in association with IRs using a well-phenotyped cellular model for methionine dependency in cancer. We performed methionine-dependency-oriented analysis by carrying out epigenome-wide conditional logistic regression analysis and combining epigenome experiments from two cancerous cell lines with mirrored methionine dependency phenotypes (i.e., MeWo-LC1 cell line being methionine dependent and which derives from the methionine-independent MeWo cell line) [30]. Interestingly, we found enrichment in epigenome signatures on loci associated with cell cycle and division, responses to IR and ultraviolet light, and DNA damage.

The present study has several strengths. First, we used an original case–control study design on several cancerous cell lines, including two cell lines known for their opposed methionine dependency phenotypes. Second, we assessed DNA epigenome signatures using the high-resolution Infinium MethylationEPIC on more than 850 k CpG probes. Third, we assessed the effect of a fractionated regimen of IRs with a cumulated dose of 10 Gy, and we assessed both baseline and end of irradiation conditions in the control arm to control for the risk of bias. Our study aimed to investigate the effect of moderate hypofractionation without generating an excess of mitotic cell death. Hypofractionation is a new standard applied in the management of prostate and breast cancers but also for radioresistant tumors such as the three cancer models investigated in the EpiMet protocol. In the irradiation protocol regimen that we applied in the present study, we aimed to model the radiation boost delivered at the end of the treatment schedule. Fourth, we report the first evidence on the association of epigenome landscape alterations with IRs, considering the methionine dependency phenotype. Fifth, we performed several bioinformatics approaches that unraveled shared enriched annotation pathways in relation to IRs exposure. Nevertheless, we acknowledge several potential limitations of the study that should be considered in interpreting our results. First, even if a fractionated regimen of IRs seems to be the more adapted experimental design to assess the effect of adaptive epigenome alterations to radiation-induced cellular stress, it remains that a higher cumulative dose (> 10 Gy) could induce a more striking epigenome signature associated with IRs and should be investigated in future experiments. Second, we did not assess and quantify the effect of IRs at the cellular level by using a surrogate biomarker to estimate DNA damage [49]. Third, even if our hypothesis on epigenome modifications in the setting of IR exposure was based on DNA methylation alterations, the role of histone modification and chromatin dynamics (e.g., H3K4me3 signature) could not be ruled out and deserves further investigation [50].

In our study, we did not perform methylation profiles on human cancer samples before and after IR exposure for several reasons. First, the EpiMet project received regulatory authorizations from the ethics committee of the University of Lorraine to perform analyses on four cell lines representative of three cancer models (HCC, melanoma, and glioblastoma). Second, we developed the EpiMet project to assess the influence of the methionine dependency phenotype on the effect of IRs on the DNA methylome landscape. Methionine dependency is defined as the ‘inability of cells to grow when methionine is replaced in culture medium by its metabolic precursor homocysteine’ [6]. According to this definition, establishing a methionine dependency phenotype is possible through in vitro testing using cell culture models and methionine-free culture media. In vivo strategies of methionine restriction are not efficient and decrease serum methionine by only 40 to 50% [51], which is not sufficient for obtaining complete methionine deprivation. In this setting, the use of methioninase-based strategies for methionine deprivation on xenografted nude mice models could be considered as part of a future development of our research framework [51, 52]. Third, according to international guidelines, IRs are performed after surgery or in definitive intent in glioblastoma, melanoma, or HCC. Thus, the interventions to collect fresh tissues of irradiated tumors would be difficult to defend in an interventional clinical trial.

In conclusion, the present study unraveled shared epigenomic adaptive mechanisms in response to IRs using in vitro models of epidemiologically leading cancerous diseases. These mechanisms are related to cell cycle and division, responses to IR, sustained angiogenesis, tissue invasion, and metastasis. Our results pave the way toward a future research agenda regarding the epigenomic correlates of radioresistance, the development of a clonal selection of radiation-resistant cells, and tumor relapse. Future experimental designs will help better understand the long-term effect of chronic cellular stress on initiating and maintaining epigenetic modifications that could initiate and perpetuate a radioresistance phenotype and its interaction with methionine dependency as a hallmark of metabolic adaptation in cancer.

## Supplementary Information

**Additional file 1: Figure S1**. Quality control regarding the genome-wide distribution of the CpG probes according to their β value. All DNA methylome profiles had a beta distribution and passed the quality criteria.

**Additional file 2: Figure S2**. 3-D plot using the three top eigenvectors (EV1, EV2, EV3) derived from the primary component analysis on the genome-wide methylome landscape of the studied cell lines, according to study conditions: ‘Baseline’ and ‘End_No irradiation.’

**Additional file 3: Figure S3**. 2-D plot using the two top eigenvectors (EV1, EV2) derived from the primary component analysis on the genome-wide methylome landscape of the studied cell lines. Cell lines are indicated using elliptical shapes. Study conditions are indicated using the color code (green: Baseline; blue: End_No irradiation; and red: End_Irradiation).

**Additional file 4:** **Table S1a.** Results from the epigenome-wide association study that assessed the effect of ionizing radiations on the DNA methylation profile comparing “End_Irradiation” vs. “End_No irradiation or Baseline” conditions.

**Additional file 5:** **Table S1b.** Results from the epigenome-wide association study that assessed the effect of ionizing radiations on the DNA methylation profile comparing End_Irradiation” vs. “End_No irradiation” conditions.

**Additional file 6:** **Table S2.** Results from the epigenome-wide association study that assessed the effect of ionizing radiations on the DNA methylation profile comparing “End_Irradiation” vs. “End_No irradiation or Baseline” conditions in the HepG2 cell line.

**Additional file 7:** **Table S3.** Results from the epigenome-wide association study that assessed the effect of ionizing radiations on the DNA methylation profile comparing “End_Irradiation” vs. “End_No irradiation or Baseline” conditions in the MeWo-LC1 cell line.

**Additional file 8: Table S4**. Results from the epigenome-wide association study that assessed the effect of ionizing radiations on the DNA methylation profile comparing “End_Irradiation” vs. “End_No irradiation or Baseline” conditions in the MeWo cell line.

**Additional file 9: Table S5**. Results from the epigenome-wide association study that assessed the effect of ionizing radiations on the DNA methylation profile comparing “End_Irradiation” vs. “End_No irradiation or Baseline” conditions in the U251 cell line.

**Additional file 10: Figure S4**. Epi-Manhattan plot reporting the results of the conditional logistic regression epigenome-wide association study that compared ‘End_Irradiation’ vs. (Baseline/End_No irradiation) according to the methionine dependency phenotype.

**Additional file 11:** **Tables S6.** Results from enrichment analyses that used the CpG probes exhibiting a difference of β values >0.1 (a) or <-0.1 (b), considering all cell lines. Results from enrichment analyses that used the CpG probes exhibiting a difference of β values >0.1 in HepG2 (c), MeWo (d), MeWo-LC1 (e), and U251 (f). Results from enrichment analyses that used the CpG probes exhibiting a difference of β values <-0.1 in HepG2 (g), MeWo (h), MeWo-LC1 (i), and U251 (j).

**Additional file 12: Figure S5**. Venn diagram illustrating the shared gene ontology annotation pathways between HepG2 and MeWo-LC1 cell lines associated with CpG probes with an increased methylation level and HepG2, MeWo-LC1, and U251 cell lines associated with CpG probes with a decreased methylation level.

**Additional file 13: Table S7.** Results from enrichment analyses that used the CpG probes with beta regression coefficient values > 200 (a) and <-200 (b) in conditional logistic regression EWAS that compared methionine-dependent MeWo-LC1 cells to their non-methionine dependent mother cells MeWo.

## Data Availability

Data are available for use in collaborative studies to researchers upon reasonable request (abderrahim.oussalah@univ-lorraine.fr; jean-louis.gueant@univ-lorraine.fr). Data will be provided following the review and approval of a research proposal (including a statistical analysis plan) and the completion of a data-sharing agreement. Responses to the request for the raw data will be judged by the IRB of INSERM UMR_S 1256.
