# Supplementary figures and images for: Ionizing radiations induce shared epigenomic signatures unraveling adaptive mechanisms of cancerous cell lines with or without methionine dependency
Source: Clin Epigenetics. 2021 Dec 1;13:212. doi: 10.1186/s13148-021-01199-y (PMC8638416; doi:10.1186/s13148-021-01199-y)

## Slide 1
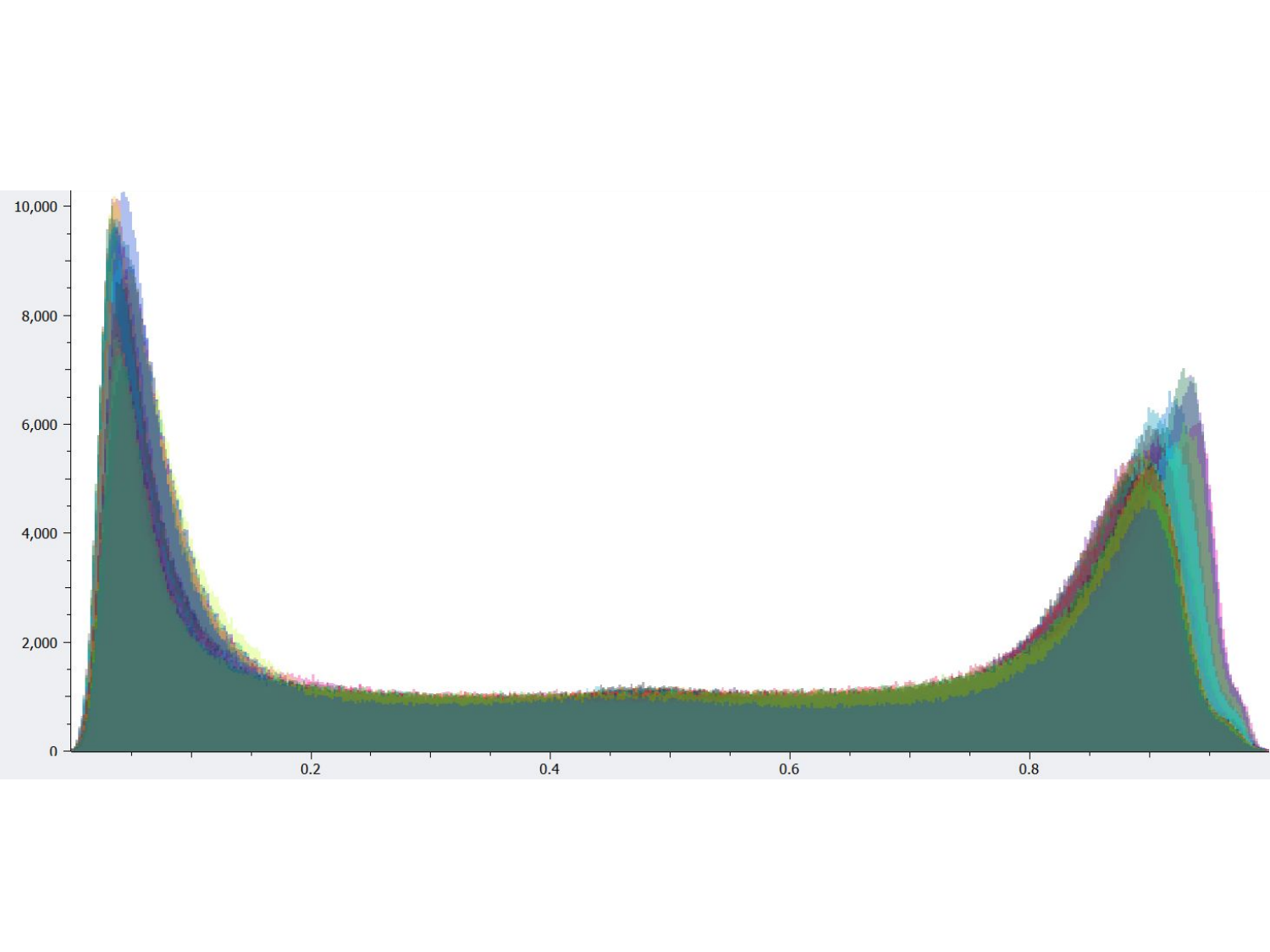

Supplement: Supplementary file 1 — Additional file 1: Figure S1. Quality control regarding the genome-wide distribution of the CpG probes according to their β value. All DNA methylome profiles had a beta distribution and passed the quality criteria. [file 13148_2021_1199_MOESM1_ESM.pptx]

## Slide 1
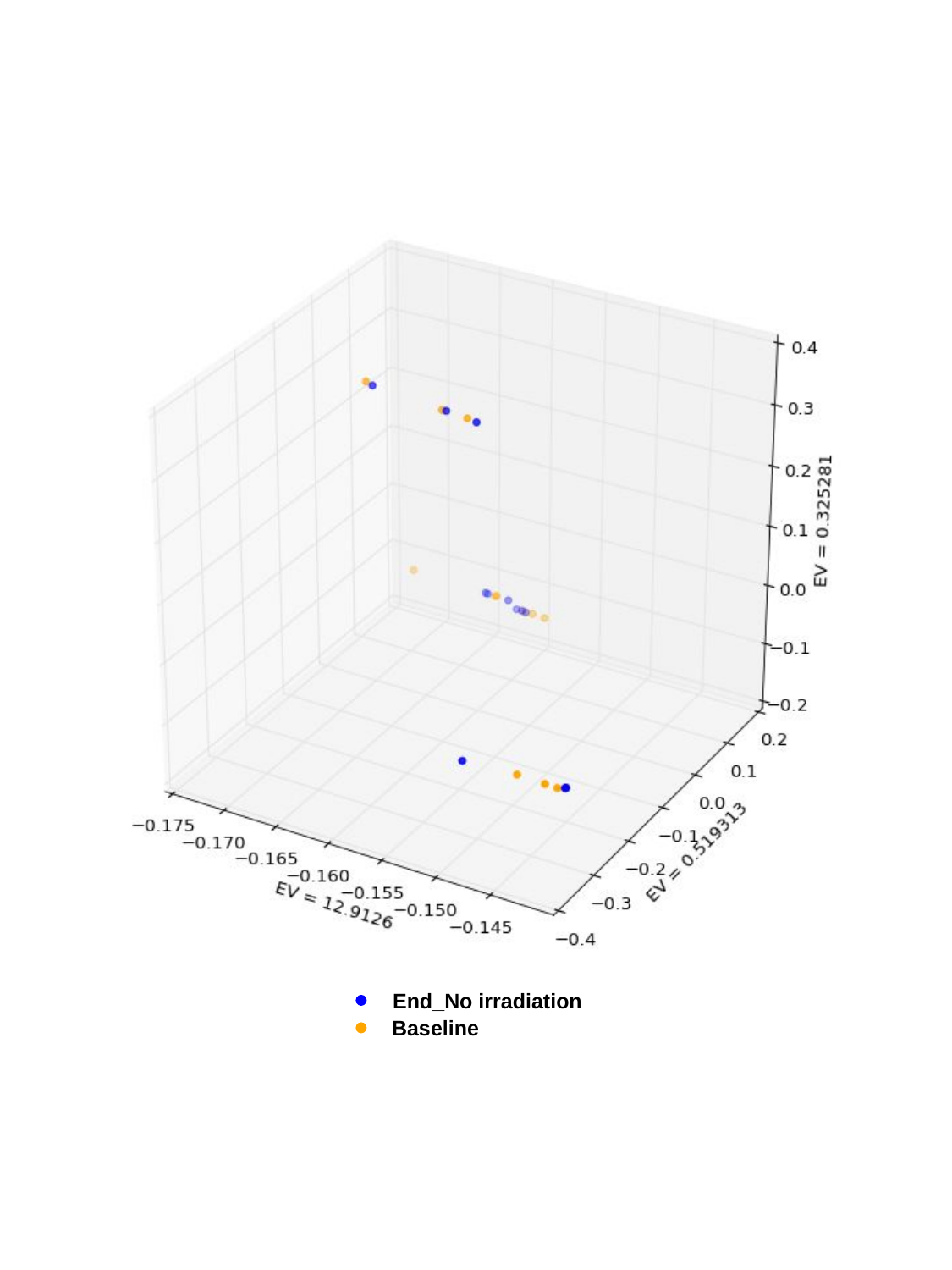

End_No irradiation
Baseline

Supplement: Supplementary file 2 — Additional file 2: Figure S2. 3-D plot using the three top eigenvectors (EV1, EV2, EV3) derived from the primary component analysis on the genome-wide methylome landscape of the studied cell lines, according to study conditions: ‘Baseline’ and ‘End_No irradiation.’ [file 13148_2021_1199_MOESM2_ESM.pptx]

## Slide 1
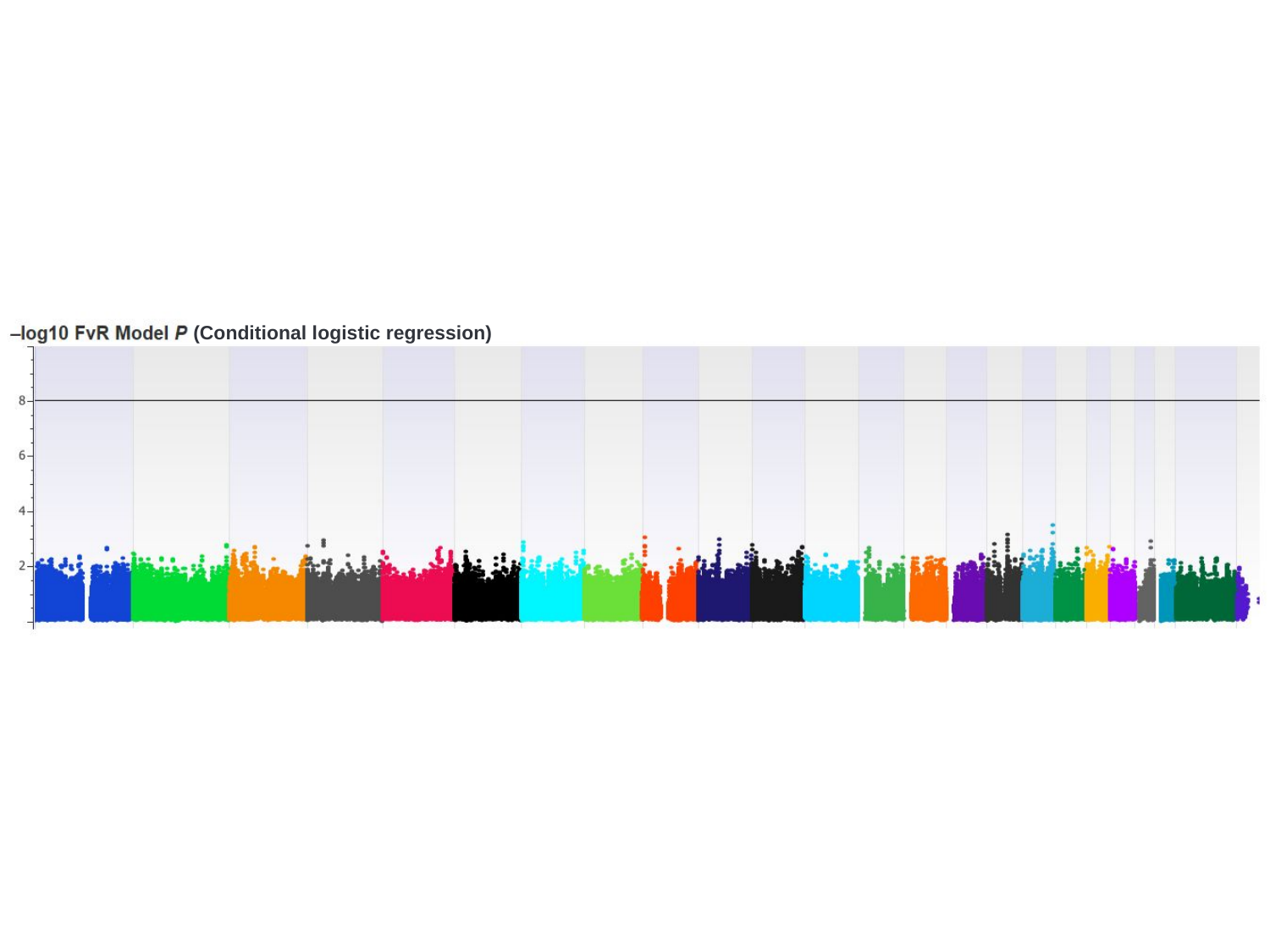

(Conditional logistic regression)

Supplement: Supplementary file 10 — Additional file 10: Figure S4. Epi-Manhattan plot reporting the results of the conditional logistic regression epigenome-wide association study that compared ‘End_Irradiation’ vs. (Baseline/End_No irradiation) according to the methionine dependency phenotype. [file 13148_2021_1199_MOESM10_ESM.pptx]
