# Supplementary material for: Ionizing radiations induce shared epigenomic signatures unraveling adaptive mechanisms of cancerous cell lines with or without methionine dependency
Source: Clin Epigenetics. 2021 Dec 1;13:212. doi: 10.1186/s13148-021-01199-y (PMC8638416; doi:10.1186/s13148-021-01199-y)
Supplement: Supplementary file 3 — Additional file 3: Figure S3. 2-D plot using the two top eigenvectors (EV1, EV2) derived from the primary component analysis on the genome-wide methylome landscape of the studied cell lines. Cell lines are indicated using elliptical shapes. Study conditions are indicated using the color code (green: Baseline; blue: End_No irradiation; and red: End_Irradiation). [file 13148_2021_1199_MOESM3_ESM.pptx]

## Slide 1
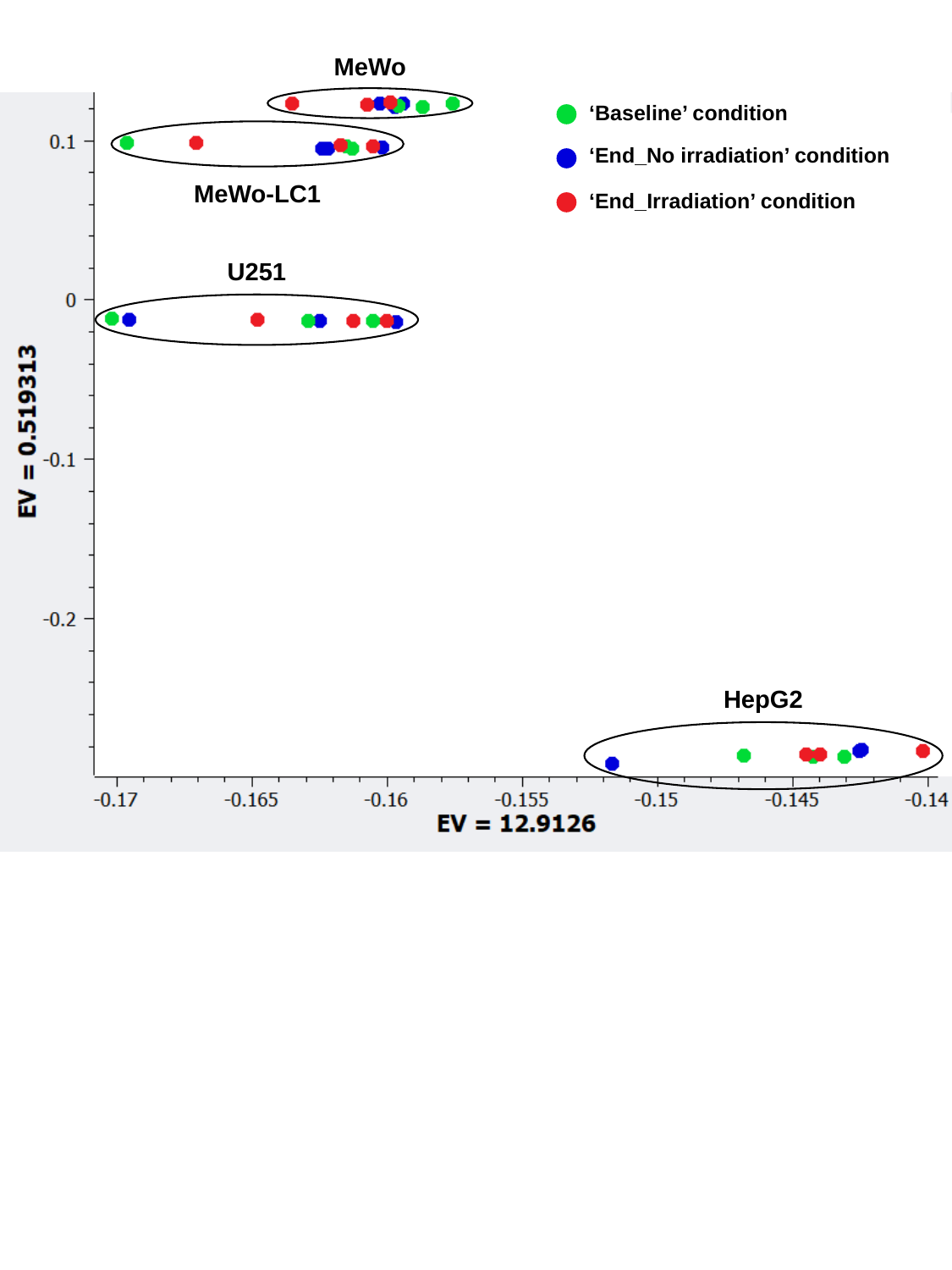

MeWo
‘Baseline’ condition
‘End_No irradiation’ condition
‘End_Irradiation’ condition
MeWo-LC1
U251
HepG2
