# Supplementary material for: Ionizing radiations induce shared epigenomic signatures unraveling adaptive mechanisms of cancerous cell lines with or without methionine dependency
Source: Clin Epigenetics. 2021 Dec 1;13:212. doi: 10.1186/s13148-021-01199-y (PMC8638416; doi:10.1186/s13148-021-01199-y)
Supplement: Supplementary file 12 — Additional file 12: Figure S5. Venn diagram illustrating the shared gene ontology annotation pathways between HepG2 and MeWo-LC1 cell lines associated with CpG probes with an increased methylation level and HepG2, MeWo-LC1, and U251 cell lines associated with CpG probes with a decreased methylation level. [file 13148_2021_1199_MOESM12_ESM.pptx]

## Slide 1
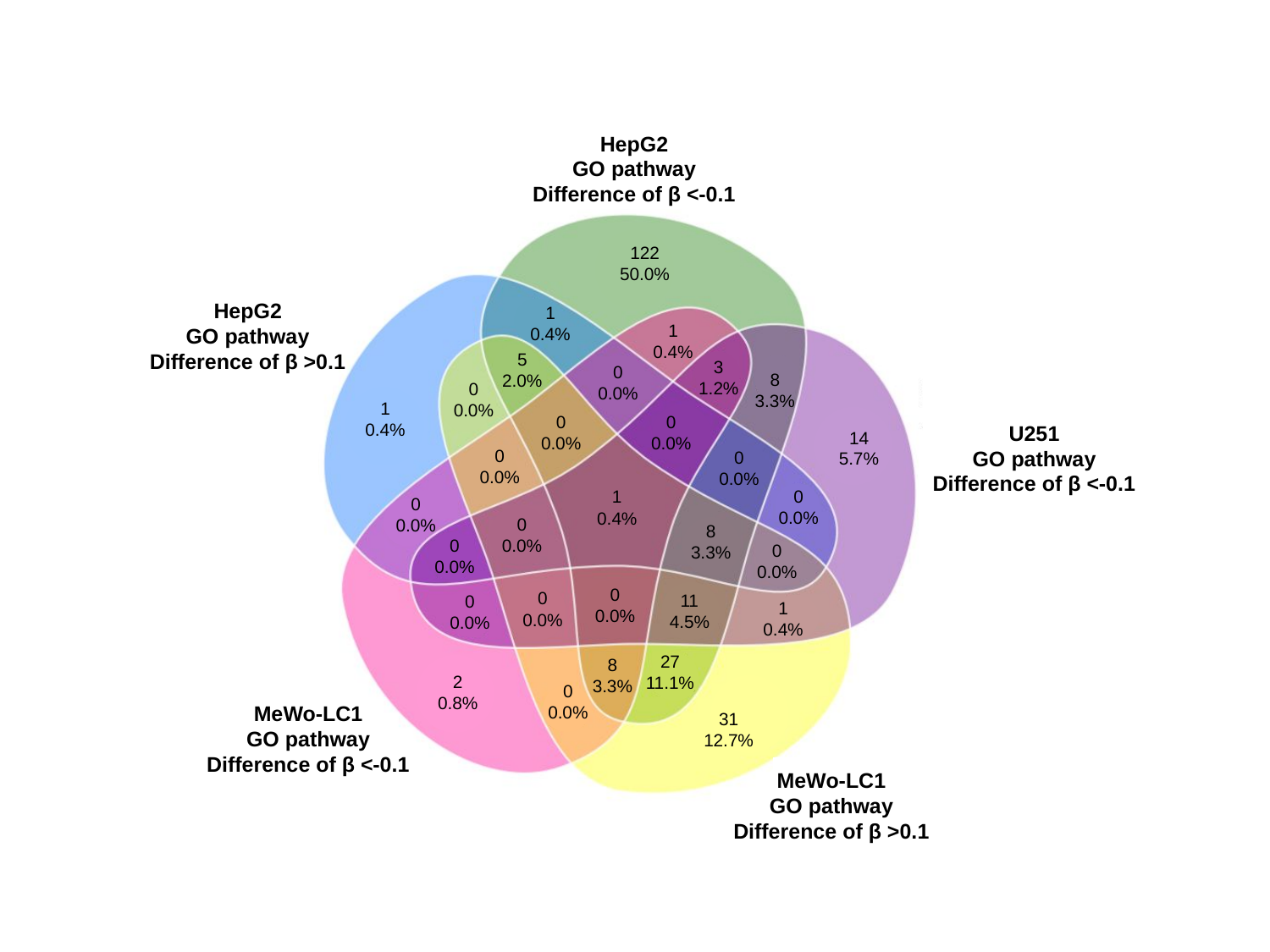

HepG2
GO pathway
Difference of β <-0.1
HepG2
GO pathway
Difference of β >0.1
U251
GO pathway
Difference of β <-0.1
MeWo-LC1
GO pathway
Difference of β <-0.1
MeWo-LC1
GO pathway
Difference of β >0.1
122
50.0%
1
0.4%
1
0.4%
5
2.0%
3
1.2%
0
0.0%
8
3.3%
0
0.0%
1
0.4%
0
0.0%
0
0.0%
14
5.7%
0
0.0%
0
0.0%
0
0.0%
1
0.4%
0
0.0%
0
0.0%
8
3.3%
0
0.0%
0
0.0%
0
0.0%
0
0.0%
11
4.5%
0
0.0%
1
0.4%
27
11.1%
8
3.3%
2
0.8%
0
0.0%
31
12.7%
